# HIV, Cancer, and the Microbiota: Common Pathways Influencing Different Diseases

**DOI:** 10.3389/fimmu.2019.01466

**Published:** 2019-06-27

**Authors:** Sabina Herrera, Javier Martínez-Sanz, Sergio Serrano-Villar

**Affiliations:** Department of Infectious Diseases, Facultad de Medicina, Hospital Universitario Ramón y Cajal, Universidad de Alcalá (IRYCIS), Madrid, Spain

**Keywords:** HIV, cancer, microbiota, immunotherapy, dysbiosis

## Abstract

HIV infection exerts profound and perhaps irreversible damage to the gut mucosal-associated lymphoid tissues, resulting in long-lasting changes in the signals required for the coordination of commensal colonization and in perturbations at the compositional and functional level of the gut microbiota. These abnormalities in gut microbial communities appear to affect clinical outcomes, including T-cell recovery, vaccine responses, HIV transmission, cardiovascular disease, and cancer pathogenesis. For example, the microbial signature associated with HIV infection has been shown to induce tryptophan catabolism, affect the butyrate synthesis pathway, impair anti-tumoral immunity and affect oxidative stress, which have also been linked to the pathogenesis of cancer. Furthermore, some of the taxa that are depleted in subjects with HIV have proved to modulate the anti-tumor efficacy of various chemotherapies and immunotherapeutic agents. The aim of this work is to provide a broad overview of recent advances in our knowledge of how HIV might affect the microbiota, with a focus on the pathways shared with cancer pathogenesis.

## Introduction

A hallmark of treated HIV infection is sustained, low-level viral inflammation. While the cause of this persistent activation of innate and adaptive immunity despite well-controlled HIV RNA replication is not completely understood, it is widely assumed that chronic defects of mucosal immunity are a major contributor (1). HIV targets the mucosa on structural and functional levels (2–4). Arguably, these disturbances will have consequences on the signals required for the coordination of commensal colonization, which may explain the shifts in microbial distributions and metabolic activity of gut microbial communities (5–7). In addition, these abnormalities caused by HIV infection have been shown to result in increased translocation of microbial products from the gut to the circulation in both animal models and HIV-infected individuals (8, 9). It has been repeatedly shown that biomarkers of bacterial translocation positively correlate with markers of T-cell activation, monocyte activation, and proinflammatory cytokines (10). It is widely accepted now that sustained low-level activation of the innate and adaptive immune systems is a major driver of AIDS and non-AIDS-related comorbidities (11–15). Collectively, these observations argue that microbial translocation, a phenomenon intrinsically linked to the gut microbiota, is a driver of inflammation, and adverse outcomes during treated HIV infection.

## Influence of the Microbiota on HIV Immunopathogenesis During Treated Infection

The gut microbiota has been associated with HIV immunopathogenesis (5, 16–19). Defining the influence of HIV on the microbiota, however, is more difficult. Studies on the impact of SIV infection in the gut microbiota of non-human primates have found only modest differences in the fecal bacterial communities between SIV-infected macaques compared to uninfected macaques, suggesting that the development of immunosuppression, rather than SIV infection itself, may drive the differences (20, 21). In addition, induction of dysbiosis with vancomycin does not accelerate the progression of untreated SIV infection (22). The effects of HIV infection on microbial diversity appear to be confounded by a number of factors, including the nadir of CD4+ T-cells (23) and the risk factor for HIV acquisition (24, 25). While admittedly there are difficulties dissecting the specific effects of HIV disease on the microbial communities, there is wide consensus that the gut microbiomes of HIV-positive individuals exhibit specific compositional and functional shifts (5, 19, 26–29). Surprisingly, the microbiota associated with HIV infection shares traits with that associated with other proinflammatory conditions, such as the depletion of butyrate-producing bacteria observed in inflammatory bowel disease (30).

**Figure 1:**
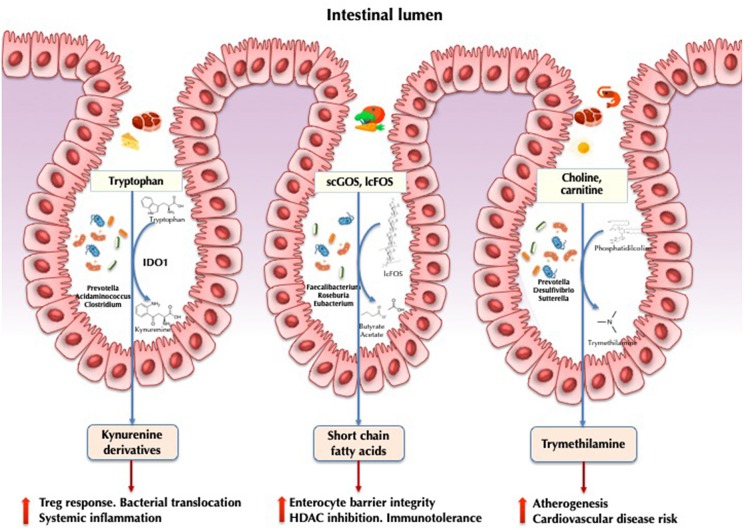
Implications of the gut microbiota in HIV pathogenesis. IDO1, indoleamine 2,3-dioxygenase 1; scGOS, short-chain galactooligosaccharides; lcFOS, long-chain fructooligosaccarides; HDAC, histone deacetylases.

It is therefore tempting to assume that so-called “HIV-associated dysbiosis” may be implicated in the sustainment of systemic inflammation in treated HIV disease. Several taxa and their associated pathways (Figure 1) have been linked with persistent immune abnormalities (5, 7, 31). The real picture, however, may be far more complex. From an ecological point of view, the components of a rapidly evolving ecosystem will respond to environmental perturbations by adapting their composition and functions to achieve the optimal fitness within their changing habitat (32). For example, the fecal microbiota of people with HIV has been shown to harbor greater abundances of genes related to resistance to oxidative stress, such as the genetic machinery for glutathione metabolism or zeatin biosynthesis pathways (7, 31).

Defining the clinical scope of the changes in gut microbial communities can be challenging because a big proportion of bacteria are dead, dormant, or inactive (33, 34). Expensive and time-consuming techniques are required to measure the proteins and metabolites synthesized by active bacteria. The extent of functional adaptation of microbial communities to the ecological perturbation induced by HIV might influence the different immunologic outcomes achieved during antiretroviral therapy (ART). In fact, HIV infection activates an important fraction of the gut microbiota. Although only 20% of the fecal microbiota is metabolically active in healthy controls, HIV infection is characterized by the activation of up to 50% of microbial communities (35). Among immunological ART responders, the metabolic activity of some taxa (Succinivibrionaceae family) is boosted, acting as anti-inflammatory buffers thanks to the accumulation of proinflammatory mediator. In addition, cannabinoid oleamide and biliverdin (a viral inhibitor) are also accumulated within bacteria and may contribute to health recovery by inhibiting viral replication, stimulating the immune system, and ultimately reducing inflammation. These findings are in sharp contrast to those observed in immunological non-responders whose gut bacteria metabolism is most similar to that of ART-naïve participants. The metabolic activity of their gut bacteria is characterized by the cleavage of the sialic and dolichol components necessary to maintain enterocyte integrity (19).

### The Kynurenine Pathway

Indoleamine-2,3-dioxygenase-1 (IDO1) involved in tryptophan catabolism via the kynurenine pathway is correlated with epithelial barrier disruption and bacterial translocation in HIV infection (36). Induction results in the production of kynurenine derivatives with immunosuppressive effects, impairing mucosal immunity, and promoting bacterial translocation and higher mortality (37). In a seminal study, Vujkovic-Cvijin et al. (5) characterized 140 genera significantly correlated with tryptophan catabolism. Some of these taxa were found to encode the genetic machinery that reproduces the same tryptophan catabolism as human IDO1. This finding was further confirmed by metabolomic analysis in gut bacteria via the detection of the kynurenine subproduct 3-hydroxyanthranilate (34). In a subsequent study combining metagenomic and metatranscriptomic data, we showed that HIV-infected individuals exhibited increased anaerobic catabolism of tryptophan via tryptophanase anaerobic fermentation compared with healthy controls (23). This expression was upregulated in the Prevotella, Acidaminococcus, and Clostridium genera. It is likely that the HIV-associated microbiota exerts a strong influence on this critical pathway at the crossroads between metabolism and immunity.

### Short-Chain Fatty Acids

Short-chain fatty acids (SCFAs) are the primary fermentation products of gut microbiota from dietary fibers. The most abundantly produced SCFAs include acetate, propionate, and butyrate (38, 39). Butyrate is a regulator of intestinal homeostasis and a modulator of immune cell response. It is involved in the maintenance of enterocyte barrier integrity and mucine production (40), induces transcription of human genes via histone deacetylase inhibition (41), and promotes immunotolerance to commensal bacteria (42). Several studies have demonstrated a decrease in butyrate-producing bacteria, including *Roseburia*, Coprococcus, Faecalibacterium, and *Eubacterium*, in both HIV-treated and ART-naïve individuals, in association with altered SCFAs profiles (17, 43). In patients with ulcerative colitis, depletion of both *Faecalibacterium prausnitzii* and *Roseburia intestinalis* has been proposed to be the hallmark of dysbiosis (44). It is increasingly accepted that the butyrate synthesis pathway supports intestinal inflammation and represents a potential therapeutic target for interventions aimed at mitigating chronic inflammation (45). Propionate and acetate have been less studied in HIV but have been linked to conferring protection against cardiovascular disease and playing other beneficial roles in other diseases (46).

### Trimethylamine-N-Oxide

Trimethylamine-N-oxide (TMAO) is a gut microbiota-dependent choline and carnitine metabolite that is responsible for an increased risk of atherogenesis and cardiovascular disease risk (47), particularly in individuals who consume large quantities of meat and possess a specific microbiome signature with enriched proportions of the genus Prevotella (48). This metabolite has also been associated with atherosclerotic plaque burden in HIV in some (49, 50) but not all (51) studies. A recent cohort study comparing the fecal microbiota of HIV-infected individuals with and without ischemic heart disease showed that high TMAO plasma levels was a marker of cardiovascular heart disease and correlated with the fecal abundance of *Phascolarctobacterium, Desulfovibrio, Sutterella*, and *Faecalibacterium* (52).

### Microbiota as a Tool for Precision Medicine for HIV

Hopefully, future studies will exploit these connections between microbiota and HIV immunopathogenesis to improve the clinical management of HIV infection. From a diagnostic point of view, one could utilize microbiota to identify individuals at higher risk of HIV acquisition (53–55), to anticipate the responsiveness to pre-exposure prophylaxis strategies with topical antiretroviral drugs (56), and to predict the risk of precancerous anal lesions (57). From a therapeutic point of view, we may gain the ability to manipulate the microbiota to enhance vaccine immunogenicity (58), boost immune recovery after ART initiation (59, 60), and attenuate chronic inflammation and bacterial translocation (61). A number of studies assessing HIV patients' dietary supplementation with prebiotics and probiotics have collectively suggested that dietary supplementation may exert some beneficial immunological effects, particularly in ART-naïve individuals (30, 59, 62–64). However, two recent controlled studies focused on ART-naive (60) and ART-suppressed (65) individuals have failed to detect significant parameters of inflammation, bacterial translocation or immune activation. These findings call into question the utility of these strategies. The first pilot study of fecal microbiota transplantation in HIV failed to demonstrate adequate engraftment of colonoscopy microbiota on the microbiota of the recipients (66). Ongoing studies (NCT02256592 and NCT03329560) are evaluating different modalities of fecal microbiota transplantation. Clinical trials assessing the use of postbiotics—metabolites or cell-wall components released by microbiota—and represent the future landscape of this fascinating field.

## Influence of Microbiota in Cancer

### Microbiota as a Trigger of Cancer Pathogenesis

Cancer is a multifaceted disease influenced by both genetic and environmental factors. Microorganisms are emerging as one of the contributors to carcinogenesis, and today we know that approximately 20% of the global cancer burden is directly attributable to infectious agents (67). Beyond the neoplasias directly linked to infectious agents, increasing evidence reveals that microbial communities as a whole play a key role in carcinogenesis by altering the balance of host cell proliferation and apoptosis; hindering anti-tumoral immunity; and influencing the metabolism of host-produced factors, ingested food components, and drugs (68, 69).

Barrier failure has been proposed to be the most relevant mechanism for bacterially driven carcinogenesis, resulting in increased host-microbiota interactions (70, 71). The failure of control mechanisms (e.g., barrier defects, immune defects, dysbiosis) is believed to represent the trigger of bacterial-driven carcinogenesis (72), leading to activation of different responses that converge in cell proliferation and cancer development. The microbiome itself represent a functional barrier by suppressing the growth of pathobionts via different mechanisms, including both resource competition and direct interference competition (73). Therefore, dysbiosis has also been associated with cancer (71). Alterations of gut bacteria have been linked to the development of colorrectal cancer (CRC) (74), but also to extraintestinal cancers, including liver (75), breast (76), and lung cancer (77, 78). While lung microbiome investigations are still in their infancy, the lung microbiotas of patients with lung cancer are distinct from those of other patients (e.g., individuals with emphysema) (79). The abundance of several types of bacteria in the lungs—including *Granulicatella, Streptococcus*, and *Veillonella*—has been proposed to be a hallmark of lung cancer (80). An association between the abundance of the Koriobacteriaceae family in the lungs and recurrence free survival has been reported (81). Furthermore, the fecal microbiota of individuals with lung cancer is depleted of Bifidobacteria (82), a commensal genus with known anti-tumoral effects. Bifidobacteria appears able to enhance the efficacy of anti-programmed cell death ligand 1 therapy (83).

### Microbiota-Associated Pathways Linked to Carcinogenesis

**Figure 2:**
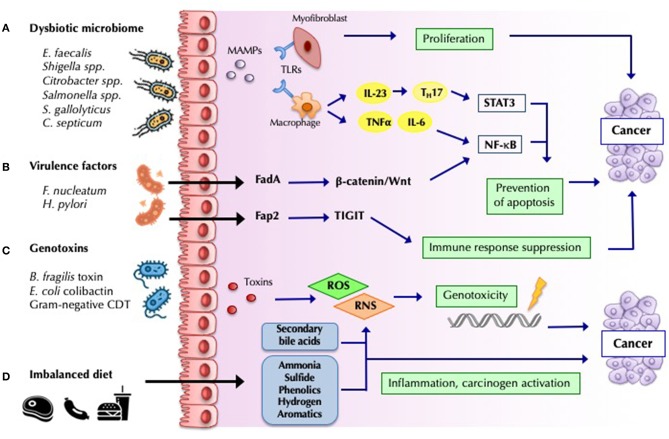
Mechanisms by which bacteria influence cancer development and progression. **(A)** Barrier loss and increased bacterial translocation engages pattern recognition by Toll-like receptors (TLRs) and activation of innate and adaptive responses. The interleukin-23 (IL-23)-IL-17 axis, IL-6, and tumor necrosis factor-α, lead to chronic inflammation mediated by nuclear factor-κB (NF-κB) and signal transducer and activator of transcription 3 (STAT3) activation, favoring tumor progression (68). **(B)** Bacterial virulence-factors promote carcinogenesis by engaging specific host pathways, which plays a decisive role in many malignancies. *Fusobacterium nucleatum* Fad-A binds host E-cadherin on colonic epithelial cells, and triggers Wnt/β-catenin pathway activation, resulting in increased NF-κB, and ultimately in increased tumor growth (84). Other virulence factor such as *H.pylori* CagA have been widely studied (68). **(C)** Some microorganisms modulate tumorigenesis through specific toxins, which induce host DNA damage. Cytolethal distending toxin (CDT) produced by Gram-negative bacteria, *Bacteroides fragilis* toxin and *Escherichia coli* colibactin constitute some of the most studied toxins identified as potential drivers of CRC (70). **(D)** Dietary residues determine the composition and metabolic activity of the microbiota. An imbalanced high-fat, high-meat, low-fiber diet, lead to a greater exposition to secondary bile acids, and protein fermentation metabolites (such as ammonia, phenols, sulfides, and nitrosamines), which have inflammatory and carcinogenic effects (85).

Recent studies of CRC have identified different mechanisms of carcinogenesis. The bacterial driver-passenger model proposes that the colonic mucosa of patients at risk of CRC is colonized by pro-inflammatory bacteria that can produce genotoxins that lead to DNA mutations and increase cell proliferation (“drivers”). These changes facilitate the replacement of the commensal bacteria with opportunistic pathogens (“passengers”) with competitive advantage in this niche, which leads to tumor progression (72). From the 1990s onward, various studies have demonstrated an association between CRC and specific colonic bacterial species, which favor the development of cancer through different pathogenic pathways (Figure 2) (86). Very impressively, *Fusobacterium nucleatum* and certain co-occurring bacteria have been found not only in primary CRC but also in distant metastases. Antibiotic treatment of mice carrying xenografts of *F. nucleatum*-positive human CRC slowed tumor growth, demonstrating the causal role of this taxon in oncogenesis (87).

Among the carcinogenic mechanisms shown in Figure 2, microbial fermentation products of dietary fiber into SCFAs, including butyrate, propionate, and acetate, with known anti-inflammatory properties (85) likely play a major role. Butyrate is one of the primary sources of energy for enterocytes, and it has been associated with the downregulation of the WNT signaling pathway, inhibition of proliferation and migration of neoplastic cells, and apoptosis induction (88). Butyrate also reinforces mucosal health via T_reg_-cell activation and IL-10 expression (89). Butyrate producers (e.g., *F. prausnitzii, Roseburia, and Bifidobacterium*) are depleted in CRC patients (69).

Another mechanism related to the catabolism of dietary precursors strongly influenced by the microbiota is the production of the proatherogenic TMAO. While the implications of this derivative of choline metabolism appear clear for cardiovascular disease (47), this pathway has been rarely studied in the field of oncology. One investigation has suggested that alterations in choline metabolism may be associated with a higher risk of CRC (90).

### The Microbiota Modulates the Efficacy and Toxicity of Anticancer Therapies

The microbiota can modulate cancer initiation and progression, but it might also influence response to therapy and treatment-related toxicity (91). First, the bioavailability of many oral drugs depends on their biotransformation in the gut by local microbiota and may also indirectly affect the metabolism of systemically delivered drugs via the regulation of xenobiotic metabolism in distant organs such as the liver (92). Second, the immune response plays an essential role in anticancer activity, and the microbiome might affect chemotherapy response via this mechanism. There is evidence that oxaliplatin and cyclophosphamide activity is modulated by gut microbiota by priming myeloid cells for high-level reactive oxygen species (ROS) production (resulting in DNA damage) and enhancing T-helper cell-mediated anti-tumor responses, respectively (93, 94). Chemotherapy-related adverse events can also be managed via microbiome modulation. For example, diarrhea caused by irinotecan toxicity, which is mediated by microbial-produced β-glucuronidases, can be regulated by targeting microbial metabolism (95). The microbiota might also play a role in response and toxicity to radiotherapy. Radiation-related mucosal injury is associated with changes in the microbiome, and germ-free mice have been shown to be resistant to radiation enteritis (91). Lastly, recent pioneering studies have yielded paradigm shifts in our knowledge of the interactions between gut bacteria and cancer therapy. The gut microbiome has been shown to modulate the anti-tumor efficacy in pre-clinical models of various chemotherapies (93, 94) and immunotherapeutic agents (96–99), including antibodies against cytotoxic T lymphocyte-associated antigen 4 (CTLA4) and anti-programmed cell death protein 1 (PD-1) (92). Individuals with metastatic melanoma responding to anti-PD-1 were enriched with *Faecalibacterium* genus in intestinal microbiota; non-responding individuals had a higher abundance of Bacteroidales (97). Another study found an abundance of *Bifidobacterium* in responding individuals; *Ruminococcus obecum* and *Roseburia intestinalis* were associated with a lack of responsiveness (99). The role of the microbiota on treatment response is further supported by striking data showing poorer survival outcomes on patients with metastatic non-small cell lung cancer or renal cell carcinoma receiving antibiotics just before or just after initiation of treatment with immune checkpoint blockade (100). Converging data support a robust interaction between specific bacteria and the systemic immune response (97–99). In subjects with non-small cell lung cancer specific memory CD4+ and CD8+ T-cells against *Akkermansia muciniphila* predicted a longer progression-free survival (98). In subjects with melanoma the abundance of *Faecalibacterium* genes positively correlated with the with a higher frequency of cytotoxic CD8 T-cell infiltration in the tumor bed. Similarly, in mice intratumoral CD8+ T-cell infiltration after anti-PD-L1 treatment correlated the microbiota composition (100).

### Is It Possible to Exploit the Microbiome to Improve Clinical Outcomes in Oncology?

Emerging evidence suggests that altering the microbiota might represent a therapeutic avenue for cancer management (101). Modulation of gut microbiota in preclinical models has been shown to enhance therapeutic response (102). Landmark studies have demonstrated that fecal microbiota transplantation from cancer patients who had responded to anti-PD-1 therapy improved the effects of PD-1 blockade in germ-free or antibiotic-treated mice (97–99). Several trials involving patients on immune checkpoint blockade undergoing fecal microbiota transplant are currently underway, but definitive data are lacking (91). Probiotics have been shown to boost anti-tumor immune responses in mice, but their off-trial use in humans is discouraged because there is still insufficient evidence to implement dietary guidelines or prebiotic administration in the setting of cancer therapy (91). Manipulation of the microbiome in cancer patients might result in novel indications for this intervention, as illustrated by the efficacy demonstrated in the first case series of patients with refractory immune checkpoint inhibitor-associated colitis successfully treated with fecal microbiota transplantation (103). Nearly 40 clinical trials assessing gut microbiota modulation in cancer are ongoing (91). The results of these investigations will inform best strategies and define indications of this therapeutic approach to improve clinical outcomes in oncology.

## HIV, Cancer, and the Microbiota: Converging Pathways and Research Avenues

Can we learn anything from microbiome studies of HIV-positive patients that may be applicable to cancer? First, the vast majority of mechanistic studies regarding the influence of the microbiome in HIV are cross sectional in nature (104). The well-known limitations of these studies are magnified by underappreciated confounding factors related to microbiota studies. For example, it took several years for the field to recognize that the increased abundance of *Prevotella* spp. observed in the first studies of HIV-infected individuals (5, 7, 16, 18) was confounded by the lower proportion of men who had sex with men in the control groups (24). Given the particular clinical profile of patients undergoing anticancer treatment, these confounders may be even more pronounced in patients with cancer.

Several pathways strongly influenced by microbiota appear to affect pathogenic mechanisms present in different conditions. Gut microbial signatures associated with clinical outcomes in both HIV and cancer and the putative mechanisms are summarized in Table 1. For example, the major butyrate producers *Faecalibacterium prausnitzii* and *Roseburia intestinalis* are depleted in subjects with HIV (17, 43), intestinal bowel disease (44), and CRC (69). Because butyrate production has been shown to promote T_reg_-cell activation and IL-10 expression (89, 105), the butyrate synthesis pathway is a potential therapeutic target for conditions in which enterocyte barrier integrity and mucosal tolerogenic immune responses are implicated. The kynurenine pathway has been also implicated in both HIV (5) and cancer pathogenesis (129). IDO1 is frequently overexpressed in many malignancies, where it correlates with poor survival and prognosis. Besides its role in immunosuppression, IDO1 promotes cancer development by inducing inflammatory neovascularization, interacting with checkpoint inhibitors, and modulating gut microbiota (130). While it is still too soon to draw conclusions about the therapeutic potential of IDO1 inhibitors for HIV disease and cancer, an increasing number of IDO1 inhibitors are currently in preclinical development or under evaluation in clinical trials (131, 132).

**Table 1:** Gut microbial signatures associated with clinical outcomes in both HIV and cancer and putative mechanisms.

**Bacteria implicated**	**Pathway/Function**	**Mechanisms**	**Biological effect**	**Clinical consequences**	**References**
↓*Faecalibacterium prausnitzii* *↓ Lachnospira* spp. *↓ Roseburia intestinalis ↓*Ruminococcaceae	SCFA-production	Histone deacetylase inhibition Human gene transcription ↓ antigen presentation ↑ immunotolerance	Immunotolerance Cell proliferation	**HIV**: systemic inflammation. Higher risk of tuberculosis **Cancer**: risk of CRD development (*Roseburia intestinalis*)	(30, 31, 97, 105, 106)
*↑ Gammaproteobacteria* *↑ Pseudomonas* spp. *↑ Bacillus* spp. *↑ Burhloderia* spp. *↑ Prevotella* *↑ Acidaminococcus*	Tryptophan catabolism	IDO1 inhibition ↑ immunosuppressive kynurenine derivatives ↓ Th17 cells	Immunotolerance Barrier failure Angiogenesis	**HIV**: bacterial translocation, inflammation, mortality **Cancer**: Overexpressed in tumoral cells (e.g., endometrial cancer, lung cancer) *IDO1 inhibitors under evaluation in both conditions*.	(5, 29, 107–109)
*↑ Bacteroides fragilis*	IL-10 signaling pathway	Polysaccharide A production TLR-2 activation IL-10 expression	Immunotolerance	**HIV**: Systemic immune activation. Periodontitis **Cancer**: anti-tumoral effects. Enhancement of CTLA-4 blockade efficacy	(5, 110–113)
↑ Actinobacteria ↓ Bacteroidetes ↑ Firmicutes ↑ Gammaproteobacteria ↑ Clostridium XIVa ↑*Faecalibacterium* spp.	Choline metabolism	TMAO production	Endothelial dysfunction Inflammation	**HIV**: carotid atherosclerosis, monocyte activation **Cancer**: malignant transformation, risk of colorectal cancer	(51, 52, 114–117)
↑ Bifidobacteria	Antitumoral immunity	↑ Dendritic cell activation ↑ CD8+ T cell priming and accumulation in the tumor microenvironment ↑ Cross-reactivity with tumor antigens	CTL responses Epithelial cell turnover Immunomodulatory strain-dependent effects	**HIV**: immune recovery under ART **Cancer**: Protection against cancer development. Enhancement of immunocheckpoint blockade efficacy.	(19, 82, 83, 118–120)
↓*Akkermansia muciniphila*	Chemotaxis	↓ Mucin degradation	Host immune regulation	**HIV**: higher systemic inflammation (sCD14, IP10) and intestinal inflammation (fecal calprotectin)**Cancer**: longer progression free-survival. Enhanced efficacy of PD-1 blockade	(26, 121–123)
*↑ Fusobacterium* spp.	Cell proliferation	TLR-4 signaling.PPAK1 cascade. Nuclear factor KB induction	Cell proliferation and oncogenesis	**HIV**: poor immune recovery after ART **Cancer**: colorrectal cancer development	(17, 124–126)
↑*Lactobacillales*	Inflammation. Antitumoral immunity	Upregulated IFN-γ, GZMB, and PRF1 expression in CD8+ T-cells	Enhanced antitumor response	**HIV**: greater immune recovery after ART **Cancer**: predictor of enhanced immunotherapy efficacy	(19, 99, 126–128)

*ART, antiretroviral therapy; CTL, cytotoxic T-cell mediated; SCFA, short-cain fatty acid; IDO1, indolamine-2,3-deoxygenase-1; LPS, lypopolisaccharide; TMAO, trimethylamine-N-oxidase*.

Analyzing gut microbiota from a functional perspective will be crucial to advancing knowledge about the role of the microbiome in the pathogenesis of cancer and understanding its interactions with immunotherapy. While bifidobacteria have not typically appeared to be compositionally relevant in most HIV studies reliant on 16S sequencing, its functional importance is clear when we assess the functional level of the microbiota. For example, while using 16S sequencing we only demonstrated modest changes in gut microbiota structure after a short prebiotic intervention, which did not include changes in the abundance of bifidobacteria (30). Using proteomics we demonstrated a 100-fold increase in the activity of the Bifidobacteriaceae family, which strongly correlated with the thymic output, a surrogate marker of the ability of the immune system to renew the T-cell pool (118). In a study aimed at identifying the bacterial biomarkers of precancerous anal lesions in HIV, *Bifidobacterium* spp. were also the most predictive taxa in stools of anal dysplasia (57). Because *Bifidobacterium* spp. enhance anti-tumor immunity and anti-PD-L1 efficacy (83), it is likely that the importance of this genus will remain underappreciated until researchers evaluate the functional level of the microbiota.

While the microbiome agenda is expanding, it is still unclear whether we can effectively manipulate the microbiome to treat HIV and cancer. Pilot studies analyzing the effects of fecal microbiota transplantation will provide powerful indications of our ability to modify clinical outcomes via microbiota manipulation. In the coming years, we look forward to learning to exploit the potential of the microbiota for precision medicine (e.g., predicting treatment responsiveness or toxicities). Gaining further insights into the mechanisms by which the microbiota influences HIV disease and cancer will help to leverage the microbiome to develop interventions for both conditions.

## Author Contributions

SS-V conceived the paper. SS-V, SH, and JM-S draft the first version of the manuscript. All authors reviewed and approved the final version.

### Conflict of Interest Statement

The authors declare that the research was conducted in the absence of any commercial or financial relationships that could be construed as a potential conflict of interest.
